# Role of oral Minocycline in acute encephalitis syndrome in India – a randomized controlled trial

**DOI:** 10.1186/s12879-016-1385-6

**Published:** 2016-02-04

**Authors:** Rashmi Kumar, Anirban Basu, Subrata Sinha, Manoj Das, Piyush Tripathi, Amita Jain, Chandrakanta Kumar, Virendra Atam, Saima Khan, Amit Shanker Singh

**Affiliations:** 1 Departments of Pediatrics, King George’s Medical University, Lucknow, UP 226003 India; 2 National Brain Research Centre, Manesar, Haryana 122051 India; 3 INCLEN Trust International, Okhla Industrial Area, Phase-1, New Delhi, 110020 India; 4 Departments of Microbiology, King George’s Medical University, Lucknow, UP 226003 India; 5 Departments of Medicine, King George’s Medical University, Lucknow, UP 226003 India

**Keywords:** randomized controlled trial, minocycline, acute encephalitis syndrome

## Abstract

**Background:**

Acute encephalitis syndrome (AES) is a public health problem in India. Neuroinfections are believed to be the most important etiology. Minocycline is a semisythetic tetracycline having excellent penetration into cerebrospinal fluid, established neuroprotective and antiviral properties besides action on nonviral causes of AES. It has been shown to be effective in animal model of Japanese encephalitis (JE). A randomized, controlled trial of nasogastric/oral minocycline in JE and AES at a single centre in Uttar Pradesh, northern India, was therefore conducted.

**Methods:**

Patients beyond 3 years of age - but excluding women aged 16–44 years - hospitalized with AES of < =7 days duration were enrolled and block randomized to receive nasogastric/oral minocycline or placebo suspension and followed up. Patients, study personnel and those entering data were blinded as to drug or placebo received. Primary outcome was cumulative mortality at 3 months from hospitalization. Analysis was by intention to treat.

**Results:**

281 patients were enrolled, 140 received drug and 141 placebo. While there was no overall statistically significant difference in 3 month mortality between drug and placebo groups [RR = 0 · 83 (0 · 6-1 · 1)], there were encouraging trends in patients older than 12 years [RR = 0.70 (0.41-1.18)] and in Glasgow Outcome Score (GOS) at 3 months (χ^2^ = 7 · 44, p = 0 · 059). These trends were further accentuated if patients dying within one day of reaching hospital were excluded [OR for 3 month mortality =0 · 70 (0 · 46-1 · 07), p = 0.090; 3 month GOS p = 0 · 028].

**Conclusions:**

A trend towards better outcomes was observed with minocycline, especially in those patients who survived the initial day in hospital. These findings should form the basis for planning a larger study and possibly including minocycline in the initial management of AES as seen here.

**Trial registration:**

The trial was registered with Clinical Trials Registry of India (CTRI) - CTRI/2010/091/006143

## Background

Acute onset of fever with alteration in consciousness is an important cause of hospital admissions in large parts of India. Such a presentation is most commonly caused by invasion of the brain by an infectious agent – virus, bacteria, protozoa, rickettsiae, mycoplasma etc. Other causes include noninfectious brain inflammations, infectious encephalopathies and other functional and structural brain disorders if associated with fever due to another cause. In 2006, the World Health Organization (WHO) coined the term ‘acute encephalitis syndrome’ (AES) for surveillance purposes [1]. AES is a symptom complex the etiology of which may change with region and time. In many parts of India, Japanese encephalitis (JE) is the most important cause of such an illness.

Uttar Pradesh is India’s most populous and one of its poorest states, with low human development indices [2]. The state is endemic for JE since the 1970s and there are annual outbreaks of this illness in the monsoon/ postmonsoon season in its eastern districts. In 2005 a severe epidemic of JE occurred here [3], after which the Government of India started a vaccination drive using the Chinese live attenuated vaccine (SA-14-14-2 strain) [4]. Even so our hospital continues to receive 1000–1200 patients of AES annually, some of which are proven to be JE. An etiological diagnosis is not reached in most patients. Treatment is mainly supportive, intravenous ceftriaxone is usually used and empirical acyclovir used occasionally.

The drug Minocycline is a semisynthetic tetracycline. Its use has been especially directed towards the central nervous system because of its lipophilic nature and ability to cross the blood brain barrier. Antibiotic action is through interference with 30-S ribosome. Yrjanheikki et al. (1998) demonstrated that it was neuroprotective in animal model of ischemia [5]. Its neuroprotective properties have since been demonstrated in diverse central nervous system (CNS) injuries including some viral encephalitides. Protective effect has been seen against hypoxic injury [6], ischemic stroke [7], amyotrophic lateral sclerosis [8], traumatic spinal cord injury [9–11], multiple sclerosis [12], Parkinson’s disease [13], Huntington’s disease [14] and diabetic retinopathy [15]. In a murine model of reovirus encephalitis, minocycline delayed disease onset and progression. Virus-induced CNS injury, apoptosis, virus titre and antigen expression were significantly decreased in the brains of minocycline-treated mice [16, 17]. In an *in vivo* simian immunodeficiency virus model of HIV CNS disease, minocycline reduced the severity of encephalitis, suppressed viral load in the brain and decreased the expression of CNS inflammatory markers [18]. In an in vitro model, effect of tetracyclines on replication of West Nile virus (WNV) was studied by cytopathic effects and virus yield reduction assay. Minocycline exerted the strongest anti-WNV activity [19].

Mishra and Basu (2008) investigated the effect of minocycline in an experimental mouse model of JE. Intravenous inoculation of GP78 strain of JEV in adult mice resulted in lethal encephalitis. Minocycline conferred complete protection in mice following JEV infection (p < 0.0001). Neuronal apoptosis, microglial activation, active caspase activity, proinflammary mediators and viral titres were markedly decreased in minocycline treated JEV infected mice on ninth day post infection. These authors concluded that minocycline was a candidate to consider in human clinical trials in JE [20–22]. The drug is already available for human use and is relatively safe. We therefore proposed a randomized, controlled trial to study the efficacy of minocycline in JE and AES. The rationale for its use in AES was threefold: firstly its neuroprotective properties, secondly its antiviral properties and thirdly its action against nonviral agents like rickettsiae and Mycoplasma which may well contribute to AES in this region. To the best of our knowledge this is the first trial of minocycline in AES.

## Methods

A parallel randomized double blind placebo controlled trial with allocation ratio of 1:1 was planned at a single site.

### Study site

The study was based in the pediatric and adult medicine wards of King George’s Medical University (KGMU) hospital in Lucknow - the capital city of Uttar Pradesh. This is a teaching hospital which caters mostly to the poor and seriously ill from the city and surrounding districts extending upto Nepal.

Prior approval for the trial was obtained from the Institutional Ethics Committee and Drug Controller General of India (DCGI). Consent and adverse event forms were filled up for each patient. A Data Safety Monitoring Board (DSMB) with nine members was formed.

### Drug and placebo

Minocycline hydrochloride and placebo powder for suspension were obtained from Unimark Remedies, India in identical bottles. Placebo differed from drug in the active ingredient being absent. The bottles were supplied in lots – four bottles/lot for children upto 12 years old and three bottles/lot for older patients. All the bottle lots were labeled individually at INCLEN before sending to the study site for use. The bottles bearing same lot ID were used for only one patient.

### Randomisation & masking

The bottles of minocycline drug and placebo powder were supplied to a 3^rd^ party- an independent member from International Clinical Epidemiology Network (INCLEN), New Delhi, who generated the randomization list with blocks of four. Separate randomization was done for children aged < =12 years and older patients.

Contents of bottles (appearance, colour, smell) were similar. The randomization sequence and code of the bottle lots were kept in safe custody by INCLEN. The patient, personnel assessing outcomes and person entering data were blinded as to drug or placebo received.

### Screening

From late August 2012 to May 2013, we actively screened patients hospitalized in pediatric (age < =12 years) and adult medicine wards with AES for enrollment in the study. Inclusion criteria for enrollment were i) age > three years but excluding women of child bearing age (between 16 and 44 years) ii) Presence of AES defined as fever with altered sensorium of < = seven days duration. Exclusion criteria were i) a firm alternative etiological diagnosis ii) consent for participation not obtained and iii) some contraindication to drug administration. Written informed consent for participation in the study was taken from the patient’s guardian. As patients were admitted, those meeting inclusion criteria without any exclusion criteria were enrolled and randomized to receive the next numbered bottle lot. No changes to eligibility criteria or outcome measures were made after commencement of the trial.

### Enrollment, treatment and work up

At admission, a detailed history, physical and neurological examination was recorded on predesigned data collection forms. Intracranial tension could not be measured but presence of hypertension with bradycardia or decerebrate posturing or Cheyne Stokes type of breathing were taken as suggestive of raised intracranial tension. A standardized work up of patients of AES as used here was adopted - including blood counts, smear and rapid test for malaria, blood urea, blood culture, serum electrolytes, creatinine and liver function tests. Neuroimaging was done whenever possible. Cerebrospinal fluid (CSF) if obtained by lumbar puncture by the treating team was examined for cell count, sugar, protein, gram stain and bacterial culture. Supportive treatment with antipyretics, anticonvulsants, nursing care, intravenous fluids, cerebral dehydrants, oxygen , ventilation and vasopressors as needed was given to patients. Intravenous ceftriaxone was also usually administered for 7–10 days. Minocycline (or placebo) suspension was reconstituted and given through nasogastric tube for seven days at a loading dose of 5 mg/kg/day followed by 2 · 5 mg/kg 12 hourly in children upto 12 years old and 200 mg loading dose followed by 100 mg 12 hourly in older patients. The agent was started by the study personnel soon after the patient was enrolled and randomized. Code on the bottle was recorded. Every dose administered was entered on the case sheet and signed. If a dose was vomited out within half hour it was repeated. If an alternative diagnosis was made after starting the agent, it was continued but specific treatment was also given. Even if the agent was withdrawn due to some contraindication later on, the patient was followed up for outcome. Contraindications were abnormal liver enzymes greater than five times normal or abnormal renal function with blood urea exceeding 35 mmol/l, diarrhea, rash or severe gastric bleeding.

### ELISA tests

CSF and serum specimen were stored at 4 C and transported in ice to the Virology Laboratory, KGMU. IgM against JEV in CSF was tested by IgM Capture ELISA (MAC ELISA) using the National Institute of Virology (Pune) kit [23] and in serum, by the Panbio Combo kit (Australia). If a specimen collected within one week of illness onset was negative, the test was repeated in serum after an interval of 7–10 days.

### Follow up and outcome

Patients were followed up daily till they left the hospital. Blood counts, liver and renal function tests were repeated after one week or earlier if indicated. Time taken to become convulsion free, afebrile, to start oral feeds and total hospital stay were recorded. Generally patients were discharged once they could be fed orally and were afebrile.

Outcome in hospital was classified as discharge or death. In many cases, the family of the patient preferred to take the patient home once the outcome appeared hopeless. This was called “leave against medical advice” or LAMA. Patients who just left the hospital without informing hospital staff were called ‘absconders’. Families of discharged patients were given appointments to revisit the hospital.

Three months after, the families were contacted on mobile phone and health of their patient was inquired into. The interviewer was unaware of the agent (drug/ placebo) received by the patient. Primary outcome in the study was cumulative mortality at 3 months from hospitalization. If the patient was alive, special questionnaires were administered telephonically. Questions included ability to walk, talk, epilepsy, mental functions, vision, hearing, focal deficits, rigidity, and abnormal movements. Overall outcome at 3 months was classified as normal/near normal, independent functioning, dependent, vegetative, or death as per the Glasgow Outcome Scale (GOS) [24]. If the patient revisited the hospital, GOS was again assessed and full neurological examination done. In some patients who could not be contacted, home visits were made.

### Power

AES mortality in our hospital is 30 % (unpublished data). To detect lowering of mortality to 15 % with 95 % confidence, with sample of 135 patients per arm, power of the study came to roughly 80 % [25].

### Adverse events

Standardized adverse event forms were filled up for each patient. Serious adverse events were reported within 24 hours and agent was discontinued.

### Data management and analysis

Double data entry followed by data matching was done. Analysis was done by the 3^rd^ party (INCLEN). Secondary outcome factors were days to become convulsion free, days to become afebrile, days to start feeding orally, duration of hospital stay in days and GOS at three months. Means were compared by ANOVA/student ‘t’ test and proportions by χ [2] test using Epi-info 3 · 5 · 4 software. Relative risks for death was calculated. Interim analysis was planned after each season and criteria to decode was observation of significant difference in primary outcome between the two groups. Kaplan Meier survival curves were generated. Analysis was by intention to treat.

### Quality assurance

Study personnel were given training in study procedures. Standard Operating Procedures were followed. A clinical research organization was hired.

## Results

Over the study period of 8 ½ months from 29^th^ August 2012 to 15^th^ May 2013, a total of 394 patients with AES fulfilled our inclusion criteria. As has been seen in the past [26, 27], patients of AES had a short history of abrupt onset of fever, often with headache and vomiting. Within a few hours to days, convulsions also occurred, after which the patient lapsed into coma. At this point the families sought hospitalization, but by the time they reached the hospital many were hemodynamically unstable. Some of our patients had rash, splenomegaly, bleeding manifestations, swelling of the body or deranged liver functions (Table 1).

**Table 1:** Baseline comparison of drug and placebo groups

Variable	Drug (n = 140)		Placebo (n = 141)	
	N/ Mean	% / SD	N/ Mean	% / SD
Mean Age^1^	15 · 4	15 · 7	16 · 1	17 · 6
Male sex^2^	102	72 · 8	108	76 · 6
Mean weight for age^1^	73 · 6	15 · 3	75 · 92	17 · 62
Mean duration of illness at presentation^1^	5 · 3	1 · 8	5 · 3	1 · 9
Headache^2^	66	47 · 1	62	43 · 9
Convulsion^2^	102	72 · 8	105	74 · 4
Mean GCS at admission^1^	8 · 6	2 · 6	8 · 2	2 · 5
Rash^2^	21	15.0	16	11.3
Swelling^2^	25	17.8	19	13.5
Bleeding^2^	5	3.5	13	9.2
Spleen palpable^2^	9	6.4	14	9.9
Meningeal signs^2^	63	45 · 0	58	41 · 1
Focal deficit^2^	11	7 · 8	17	12 · 0
↑ Tone^2^	65	46.4	67	47.5
↑ ICT^2^	13	9 · 3	20	14 · 2
Decerebration^2^	8	5 · 7	13	9.2
Mean Hb (gm/l) ^1^	105	20	106	24
Mean TLC^1^ (x 10^9^/ l)	12.5	8.6	13.6	8.2
Mean polymorph %^1^	69 · 13	13 · 65	70 · 29	14 · 9
Mean platelets^1^(x 10^9^/ l)	143 · 3	95 · 4	166 · 2	214 · 8
Mean CSF protein^1^ (g/l)	0 · 80	0 · 78	0 · 91	1 · 3
Mean CSF/ blood sugar ratio^1^	0 · 56	0. ·2	0 · 55	0 · 21
Mean CSF cells (per cu mm) ^1^	114 · 2	304 · 2	283 · 1	1118 · 7
Mean sAST^1^ (u/l)	116 · 6	149 · 5	132 · 8	244 · 2
Mean sALT^1^(u/l)	103 · 8	203 · 4	124 · 5	355 · 3
Mean s Protein (g/l) ^1^	74	68	77	79
Mean s Albumin (g/l) ^1^	34	07	32	07
Mean s Urea^1^ (mmol/l)	15 · 2	8 · 6	17 · 3	11 · 1
Mean s creatinine^1^ (μmol/l)	97 · 2	79 · 6	106 · 1	79 · 6

^1^ Comparison of means by 2 sample t test; ^2^ comparison of proportions by Chi square test; GCS: Glasgow Coma score; ICT: intracranial tension; Hb: hemoglobin; TLC: total leucocyte count; CSF: Cerebrospinal fluid; AST: aspartate transaminase; ALT: alanine transaminase; none of the differences are significant at p < 0 · 05

**Fig. 1:**
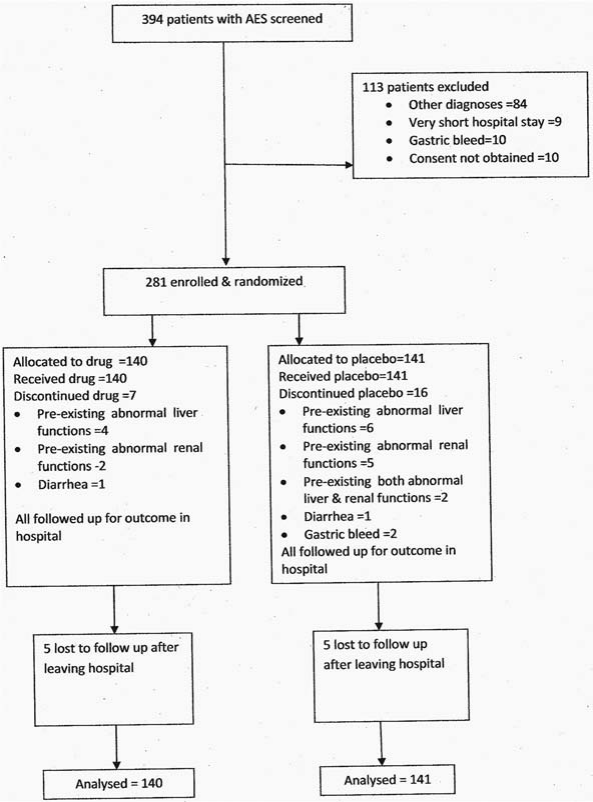
Flow Diagram showing enrollment and follow up in the two arms of the study

Of the 394 patients seen, 113 were excluded and 281 were enrolled. Reasons for exclusion were : other diagnoses –84; consent not obtained –10; very short hospital stay –9 and gastric bleeding −10. Lumbar puncture was done in 244 patients. Other diagnoses reached were hepatic encephalopathy –48; uremic encephalopathy −5; bacterial meningitis –6; tuberculous meningitis –5; head injury –3; febrile convulsions −5; other seizure disorder- 12. Figure 1 shows the flow diagram of patients in the two arms,

Only 29 patients tested positive for JE. This number was too small for a separate analysis with power of 0.3 only. One hundred and forty patients received drug and 141 received placebo. There were no significant baseline differences between the two groups (Table 1).

The trial was stalled in June 2013 after nine months following new requirements issued by the Drug Controller General of India who notified modified guidelines in 2013 (after the commencement of this trial), regarding the conduct of drug trials in India. These importantly included guidelines regarding compensation of those on the treatment arm as well as in the placebo arm. Because we were not sure of the implications of the modified regulations in a disease with high intrinsic mortality and long term morbidity as AES, the trial was stopped.

A total of 19 patients had some contraindication in the form of pre-existing deranged liver (10) or renal functions (7) or both (2). The results of these investigations were received after the agent had been started. In four patients there were untoward events *after* the agent was started (adverse events) - severe gastric bleed and diarrhea in two patients each. The agent was withdrawn in all these. There was no significant difference between drug and placebo groups in the frequency of adverse events.

Of the total 281 patients, 57 died in hospital, 36 left against medical advice (LAMA), 6 absconded and 182 were discharged. Of the 57 patients who died in hospital, 27 (47 · 4 %) died within one day, 32 (56 · 1 %) within 2 days and 42 (73 · 7 %) within three days. Of the 224 patients who left the hospital alive, 214 could be followed up for ascertainment of mortality at three months – 179 by phone alone, 33 by hospital visit and two by home visit. There was no significant difference between treatment groups in type of follow up. Of the 188 discharged or absconded patients , 171 were alive, nine were dead at three months, and eight could not be followed up. Of the 36 patients who left against medical advice, 29 were dead at three months, five were alive, and two could not be followed up. Cumulative mortality at three months was 95/271 (35 · 1 %).

**Fig. 2:**
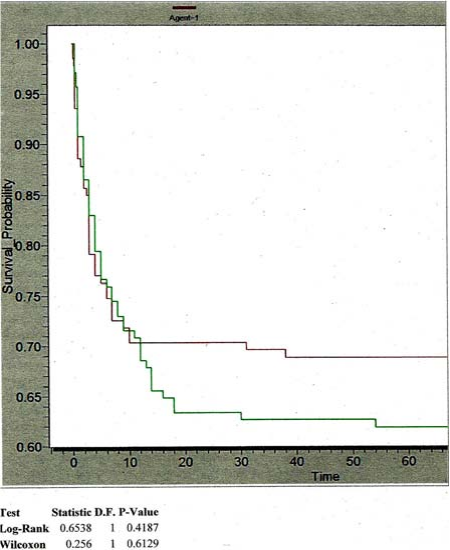
Kaplan Meier Survival curves in the two groups. Agent 1 (darker line) = Drug

The cumulative three month mortality according to drug or placebo received shows no significant difference (Table 2). Separate analysis was done for children upto 12 years of age and older patients and if all those lost to follow up were considered dead or alive but again there are no significant differences (Table 2), though those over 12 years fared better (RR 0 · 7, CI 0 · 4 – 1 · 2, p = 0 · 173). Figure 2 shows the Kaplan Meier survival graphs.

**Table 2:** Comparison of 3 month mortality in drug and placebo groups

3 month mortality	Drug (N = 140)		Placebo (N = 141)		RR (95 % CI) for death with Drug	P*
	N	%	N	%		
Dead	43	30·7	52	36·9	0·83 (0·60-1·15)	0·270
Alive	92	65·7	84	59·5		
untraced	5	3·6	5	3·6		
Age > 12 years (n = 100)	N = 50		N = 50			
Dead	15	30·0	21	42·0	0·70 (0·41-1·18)	0·173
Alive	32	64·0	25	50·0		
untraced	3	6·0	4	8·0		
Age < =12 years						
(n = 181)	N = 90		N = 91			
Dead	28	31·1	31	34·1	0·92 (0·61-1·40)	0·709
Alive	60	66·6	59	63·7		
untraced	2	2·2	1	1·1		
Considering that all those lost to follow up were dead at 3 months						
Dead	48	34·3	57	40·4	0·85 (0·63-1·15)	0·345
Alive	92	65·7	84	59·6		
Considering that all those lost to follow up were alive at 3 months						
Dead	43	30·7	52	36·9	0·83 (0·60-1·16)	0 · 274
Alive	97	69·3	89	63·1		
JE IgM positive patients (29)						
Dead (7)	2	28.6	5	71.4	0.33 (0.03-2.72)	0.384
Alive (20)	11	55.0	9	45.0		
untraced (2)	2	28.6	0	0		

*Chi square test RR: relative risk 95 % CI: 95 % confidence intervals

When the Glasgow Outcome Score at three months was compared between treatment groups, the difference approached significance with better outcome with drug ( p = 0 · 059) (Table 3).

**Table 3:** Comparison of secondary outcomes in drug and placebo groups

Outcome	Drug (140)		Placebo (141)		P
	N/Mean	%/SD	N/Mean	%/SD	
Discharged^2^	92	65 · 7	90	63 · 8	0 · 740
Absconded^2^	3	2 · 1	3	2 · 1	0 · 992
LAMA^2^	14	10 · 0	22	15 · 6	0 · 159
Death in hospital^2^	31	22 · 1	26	18 · 4	0 · 440
Mean days to become afebrile^1^	3 · 63	2 · 94	3.72	2 · 53	0 · 926
Mean days to become convulsion free^1^	1 · 35	1 · 14	1.22	0 · 87	0 · 411
Mean days to full consciousness^1^	3 · 37	2 · 6	3.06	2 · 19	0 · 399
Mean days to oral feeds^1^	3 · 06	2 · 71	3 · 21	2 · 85	0 · 702
Mean days till discharge^1^	6 · 86	4 · 97	6 · 66	4 · 39	0 · 721
Glasgow Outcome scale at 3 months^2^ Normal/near normal Independent Dependent Vegetative Dead	75 3 14 0 43	53 · 6 2 · 1 10 · 0 0.0 30 · 7	60 11 11 0 52	42 · 5 7 · 8 7 · 8 0.0 36 · 9	Chi Square = 7 · 44 P = 0 · 059
3 month GOS Unknown	5	3 · 5	7	4 · 9	

^1^ Comparison of means by 2 sample t test; ^2^ comparison of proportions by Chi square test; SD: standard deviation,

LAMA: left against medical advice; GOS: Glasgow Outcome score

Post hoc exploratory analyses stratifying for number of doses received and Glasgow Coma Score at admission did not reveal significant differences. However when patients dying within one day of admission were excluded, there was significantly better overall outcome at three months in those receiving minocycline (p = 0 · 028) and also a trend towards lower cumulative 3 month mortality (p = 0.090) (Tables 4 and 5).

**Table 4:** Comparison of 3 month mortality in drug and placebo groups in patients surviving initial day (N = 251)

3 month mortality	Drug (123)	Placebo (128)	RR (95 % CI)	p
Dead	27	40	0 · 70 (0 · 46-1 · 07)	0 · 090
Alive	91	83		
Unknown	5	5		

*Chi square test RR: relative risk 95 % CI: 95 % confidence intervals

**Table 5:** Comparison of 3 month Glasgow Outcome Scale in patients surviving initial day (N = 251)

3 month GOS	Drug (123)	Placebo (128)	p
Normal or near normal	74 (62.7)	59 (48.8)	0 · 028^1^
Independent	3 (2.5)	11 (9.1)	
Dependent	14 (11.9)	11 (9.1)	
Vegetative	0 (0)	0 (0)	
Dead	27 (22.9)	40 (33.1)	
GOS unknown	5	7	

*Chi square test for trend ^1^ Significant

## Discussion

We set out to study the effect of minocycline in JE and also AES. However, required sample size did not accrue for any meaningful analysis of JE and we present results only for all AES.

Our study setting is one of the poorest and most underdeveloped in India. This trial reflects the difficulty of conducting trials in limited resource settings such as ours, where regulatory laws are still evolving. Etiologic diagnosis of encephalitis is an arduous and costly undertaking even in more affluent settings. Although AES has a seasonal occurrence here, it may have a varied etiology and our patients of AES were likely a mixed group. Important causes in this region include JE, bacterial meningitis and dengue encephalopathy [28]. Bacterial causes are difficult to exclude because patients often have received prior treatment outside which can change the CSF findings and render the culture sterile. There are also case series of nonviral causes like rickettsioses [29] and leptospirosis [30] from various parts of the country. Mycoplasma encephalitis though difficult to diagnose may also occur [31]. In fact, ascertainment of etiology in every patient is an almost unachievable target. Therefore our approach was to conduct a trial with minocycline in AES regardless of etiology. The drug is a known neuroprotective agent, a suppressor of deleterious neuroinflammation and may act as an antiviral (as proven in animal models of viral encephalitis) besides its primary indication as an antibiotic.

We excluded children below three years because tetracyclines are contraindicated in young children. All registered trials of minocycline in conditions such as autism and fragile X syndrome included children beyond three years. Women of child bearing age were excluded because of risk of teratogenicity with unknown early pregnancy. Only patients with short duration of illness a week or less were included because the clinical course is usually already decided beyond that time.

We used nasogastric or oral minocycline as use of intravenous minocycline would be logistically difficult. Minocycline has to be diluted in 5 ml water and immediately further diluted in 500–1000 ml fluid (dextrose/normal saline/Ringers) before administration [32]. Giving such a large volume of fluid twice daily may not be feasible in a critically ill child. Nasogastric/oral route is likely to be effective as minocycline being extremely lipophilic, is almost completely absorbed after oral administration with very good CSF penetration [33]. Oral doxycycline has been used for treating intracranial infections like neurosyphilis and lyme neuroborreliosis with results equivalent to intravenous ceftriaxone [34, 35]. Minocycline is twice as lipid soluble as doxycycline with better CSF penetration [36]. We used a dose of drug which is somewhat similar to that recommended in children and adults [32] and for which safety and pharmacokinetic data are available^37^. Delivery of drug through the gastrointestinal route in a seriously sick child does not guarantee absorption. On the other hand, CSF levels may be higher in acute central nervous system infections due to disruption of blood brain barrier. Since it was not possible to measure serum or CSF levels of minocycline, more cannot be said about this. Our enrolled patients received a variable number of minocycline doses, but randomization would ensure that these differences were even between the two groups.

AES as seen here often has a compact clinical course. At first there is only fever with other nonspecific symptoms. The illness becomes clinically distinct after convulsions or coma sets in. After this deterioration and mortality may be swift. Travel to the city from far flung rural areas would be initiated only after specific symptoms appear and could take time. Thus many patients are already moribund when they reach our hospital. In such case, even if the drug is started as soon as the patient arrives, there may be no benefit because the illness has already wreaked havoc. Of the total in-hospital mortality almost half (47 · 4 %) occurred within a day of admission. We could think of no way to counter this difficulty when conducting such a trial.

In this study minocycline did not have statistically significant benefit on cumulative mortality at three months although an encouraging trend was seen in older age group beyond 12 years. Effect on overall outcome at 3 months by GOS approached significance (p = 0 · 059). However, a closer look at the data revealed that if we excluded patients who died very soon after reaching the hospital, outcomes were significantly better in minocycline treated group. Better overall three month outcome was observed in patients who survived the initial day in hospital. This indicates that the effect in the entire group was being diluted because of inclusion of already moribund and dying patients. Once these patients were excluded from the analysis, beneficial effect became evident.

## Conclusions

We conclude that there is a trend towards better outcomes with minocycline in AES seen here, especially in patients who survive the initial day in hospital. Exactly how minocycline is effective - through its neuroprotective action, antiviral/antiapoptotic effect or direct effect on nonviral agents or a combination of these, is unclear. The care taken in designing and adhering to the laid out protocol would ensure the reliability of the data. Our results would be applicable to Uttar Pradesh and large parts of eastern and southern India and possibly other regions of the world where AES has an unclear or somewhat similar etiology. Management of AES is a crucial concern in this region and given its magnitude, even a modest benefit assumes importance. These findings could form the basis for planning a larger trial using the same or different route of administration and dose of minocycline in management of AES here.

## Ethics approval

Prior approval for the trial was obtained from the Institutional Ethics Committee and Drug Controller General of India (DCGI). Consent and adverse event forms were filled up for each patient.

## Abbreviations

AES: Acute encephalitis syndrome
JE: Japanese encephalitis
CNS: central nervous system
CSF: cerebrospinal fluid
JEV: Japanese encephalitis virus
Mac ELISA: IgM Antibody capture ELISA
GCS: Glasgow coma scale
GOS: Glasgow outcome scale
